# Mechanisms of the Complex Thermo-Mechanical Behavior of Polymer Glass Across a Wide Range of Temperature Variations

**DOI:** 10.3390/polym10101153

**Published:** 2018-10-16

**Authors:** Weidong Liu, Liangchi Zhang

**Affiliations:** Laboratory for Precision and Nano Processing Technologies, School of Mechanical and Manufacturing Engineering, The University of New South Wales, New South Wales 2052, Australia; weidong.liu@unsw.edu.au

**Keywords:** polymer glass, frequency spectrum, structural relaxation, modulus

## Abstract

This paper aims to explore the mechanisms of the complex thermo-mechanical behavior of polymer glass across a wide range of temperature variations. To this end, the free vibration frequency spectrum of simply supported poly(methyl methacrylate) (PMMA) beams was thoroughly investigated with the aid of the impulse excitation technique. It was found that the amplitude ratio of the multiple peaks in the frequency spectrum is a strongly dependent on temperature, and that the peaks correspond to the multiple vibrational modes of the molecular network of PMMA. At a low temperature, the vibration is dominated by the overall microstructure of PMMA. With increasing the temperature, however, the contribution of the sub-microstructures is retarded by β relaxation. Above 80 °C, the vibration is fully dominated by the microstructure after relaxation. The relaxation time at the transition temperature is of the same order of the vibration period, confirming the contribution of β relaxation. These findings provide a precise method for establishing reliable physical-based constitutive models of polymer glass.

## 1. Introduction

The mechanical properties of polymer glasses are dependent strongly on temperature and strain rate [1,2,3,4,5]. This is due to the polymer glass’s amorphous microstructure which consists of the backbone molecular microstructure formed by the primary covalent bond and the sub-microstructures linked by the secondary bonds such as hydrogen bond and van der Waals’ force [6,7]. Under thermal and mechanical loadings, the secondary bond can be broken and the structure can be rearranged cooperatively [8,9], making the mechanical properties of polymer glasses strongly temperature and rate dependent.

The structural relaxation of polymers makes the determination of the mechanical properties of polymer glass more difficult. For example, the β-relaxation of the polymer glass PMMA (Poly(methyl methacrylate)) appears around room temperature (0 to 80 °C) [8,9]. With increasing the temperature, structure relaxation would become faster and in turn affect the conformation of polymer microstructure and its properties [8,10,11,12]. It was reported that the molecular mechanism behind the β-relaxation is due to the hindered rotation of the –COOCH_3_ group about the C–C bond that connects it to the main chain [8]. The mechanical properties of the PMMA may then be altered.

Viscoelastic-plastic constitutive models have been developed to describe the mechanical behavior of polymer glass under various loading conditions, in which modulus is one of the most important parameters [13,14,15]. However, most models are phenomenological and the parameters are obtained by the curve-fitting of experimental data. As such, the underlying physical foundation is missing. Some statistical models have also been developed to evaluate the elastic properties of polymer network such as the Rouse model and tube model [16]; however, these models mainly focus on the rubber-stage polymers (melt), and cannot predict the elastic properties of the materials below their glass transition temperatures (*T*_g_) [17,18].

Various techniques have been used to measure the moduli of polymers, including the uniaxial compressive and tension tests [19,20] and the dynamic mechanical analysis (DMA) [21,22]. For example, the Young’s modulus of polymers is determined by calculating the initial slope of a uniaxial stress-strain curve [19,20]. The DMA uses vibrational loads to excite the polymer specimen and then measure the storage modulus and loss modulus from the material’s response [21,22]. The DMA can also be used for characterizing the structural relaxation and glass transition of polymer glass. However, all the above techniques can only provide an averaged overall modulus of the material, which is not suitable for developing physics-based constitutive models.

The impulse excitation technique (IET) has been widely used for characterizing the elastic properties of advanced materials [23,24]. The advantages of this technique lie in its reliable vibrational theory background, simple set-up, and non-destructive nature. This technique is also a convenient tool to study the internal structure and property changes of materials. For example, when this method was used to study the mechanical reliability of glassy carbon at high temperature, oxidation-induced deterioration was identified by the irreversible decrease of modulus after a heating/cooling cycle [25]. When the method was used to measure the mechanical properties of borosilicate glass near *T*_g_, the structure relaxation of the material was revealed through the temperature dependence of the modulus [26]. Considering that the damping of the free vibration of a specimen is mainly attributed to the internal microstructure of its material [27], the network relaxation in a polymer glass is thus expected to affect the vibrational frequency spectrum measured by the impulse excitation technique.

In this paper, we will investigate the mechanisms of the complex thermo-mechanical behavior of PMMA across a wide range of temperature variations with the aid of impulse excitation technique. The free vibrational frequency spectrum of PMMA specimen will be analyzed during the continuous heating and cooling process from room temperature up to a maximum temperature above *T*_g_ (Glass Transition Temperature). Our goal is to explore how the structural relaxation of PMMA below *T*_g_ would affect its vibration and whether one can establish relations between microstructure and macro-scale vibrational signals.

## 2. Materials and Methods

The raw PMMA material used in this study was fabricated by Palram Australia PTY LTD (Suntuf brand). It is a flat extruded acrylic plate with >99.3 wt % Poly (Methyl Methacrylate) and <0.7 wt % Methyl Methacrylate. Several specimens with two dimensions (37 mm × 11 mm × 1.1 mm and 59 mm × 8.2 mm × 7.8 mm) were prepared for the impulse excitation tests. Before tests, all the specimens were inspected by a polariscope (Fullauto StrainEye LSM-9000W, Luceo Co., Ltd., Tokyo, Japan) and no processing-induced residual stress was found.

**Figure 1:**
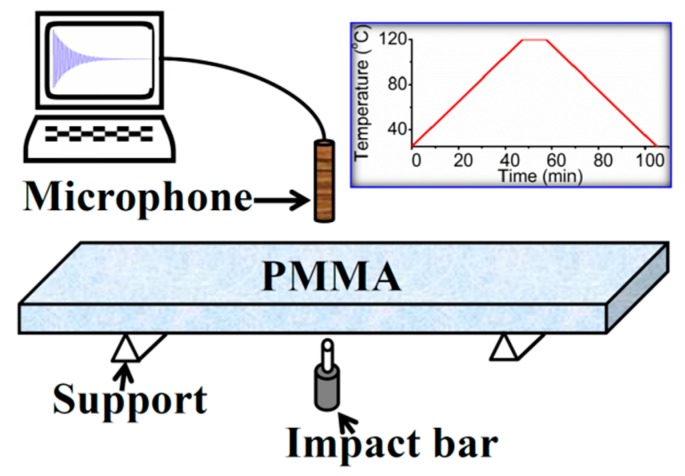
Schematic picture of the setup of impulse excitation test and the temperature profile (inset).

The tests were conducted in the furnace of IMCE RFDA 1100 HT (IMCE Inc., Genk, Belgium), in which the sample was mounted by using the set-up shown in Figure 1. During the test the sample was excited by an impact bar every 10 s. The sound produced from the vibration of the sample was transmitted along a ceramic bar and captured by a high-precision microphone outside the furnace. The recorded vibrational signal in the time domain was converted to frequency by Fast Fourier Transform (FFT). In this way, the vibration frequency spectrum of the specimen was obtained. To study the temperature effect, a heating-cooling cycle of room temperature to 120 °C was applied to a specimen, because the glass transition temperature of PMMA is 105 °C. The heating and cooling rates were 2 °C/min, and a holding time of 10 min was applied at the maximum temperature (see the inset of Figure 1). Another heating/cooling rate of 5 °C/min was also used for comparison. All the experimental results were verified by at least three repeated tests.

## 3. Results

**Figure 2:**
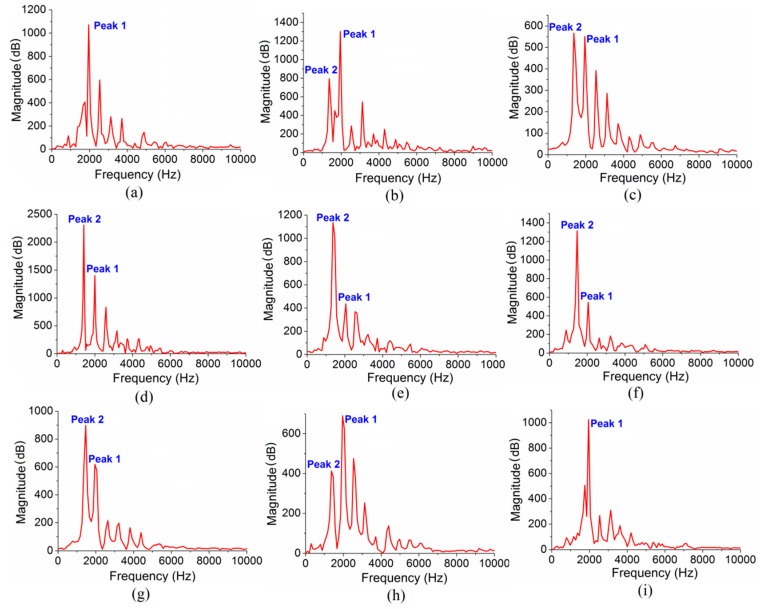
Frequency spectrum of freely vibrated poly(methyl methacrylate) (PMMA) beam (37 mm × 11 mm × 1.1 mm) during heating at (**a**) 25 °C; (**b**) 58 °C; (**c**) 75 °C; (**d**) 77 °C; (**e**) 82 °C; (**f**) 120 °C; and cooling at (**g**) 82 °C; (**h**) 65 °C; (**i**) 23 °C.

Figure 2a presents a typical frequency spectrum of the free vibration of PMMA beam (37 mm × 11 mm × 1.1 mm) at room temperature (25 °C). Different from the frequency spectrums of metals or ceramics which usually have one single sharp peak only [25,26], that of PMMA (Figure 2a) shows a series of peaks, with the highest at 1960 Hz (Peak 1). Considering that PMMA at room temperature can be taken as a solid and the dominant peak should correspond to the first-order bending vibration mode.

The first-order bending vibration of a simply supported beam, *f*, is determined by its modulus, geometry, and mass [26] as

(1) $$f=\sqrt{1.0565\frac{Ewt^{3}}{ml^{3}}}\;$$

where *E* is Young’s modulus; *m* is the specimen mass; and *l*, *w*, and *t* are the length, width, and thickness of the specimen, respectively. The Young’s modulus of PMMA at 25 °C can then be worked out as *E* = 6.43 GPa by using Equation (1) with the dominant frequency available (1960 Hz). With increasing the temperature, however, a peak (Peak 2) with a smaller frequency (1404 Hz at 58 °C) starts to increase, as shown in Figure 2b. At 75 °C (Figure 2c), the frequency of Peak 2 increases to 1413 Hz, and its amplitude almost reaches that of Peak 1. At 77 °C (Figure 2d), the amplitude of Peak 2 becomes significantly larger than that of Peak 1. At 82 °C (Figure 2e), Peak 2 becomes much higher than the others and dominates at higher temperatures up to 120 °C (Figure 2f). Beyond 120 °C, the specimen became soft quickly and no reliable signal can be obtained. In cooling, the amplitude of Peak 2 decreases while the amplitude of Peak 1 increases. The changeover happens in 75–65 °C and finally Peak 1 dominates the spectrum again at room temperature. The spectrum variation has been found to be similar to the above when a higher heating/cooling rate (5 °C/min) is applied. The possible vibrational mode of Peak 2 will be discussed in Section 4.

**Figure 3:**
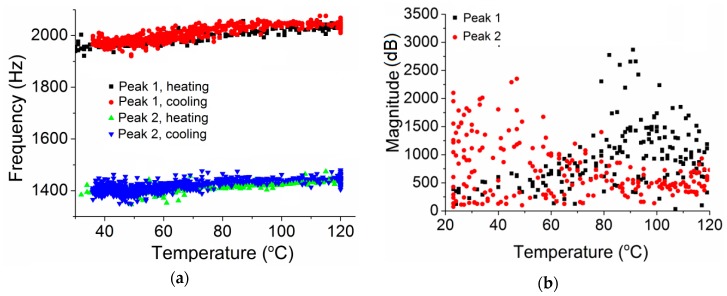

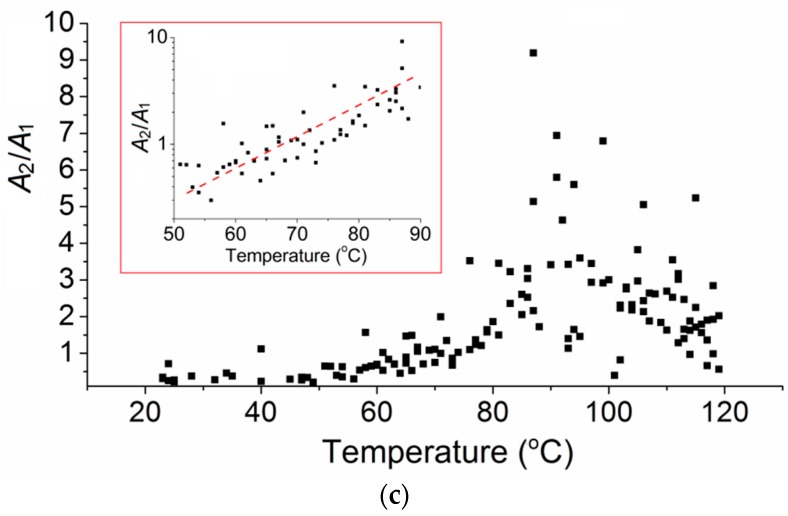
(**a**) The frequency changes of Peak 1 and Peak 2 with temperature; (**b**) the magnitude changes of Peak 1 and Peak 2 with temperature; and (**c**) the magnitude ratio (*A*_2_/*A*_1_) change with temperature (the inset is with logarithmic vertical scale).

Figure 3a shows the frequency changes of Peaks 1 and 2 with temperature. It is noted that the frequencies of both peaks increase with temperature increment, and are reversible during cooling. Figure 3b presents the magnitude changes of the two peaks. It is clear that the changeover occurs in the range of 50 and 80 °C. The scattering is caused by the difference of the impact load applied each time, making the magnitude of the vibration ununiform. Figure 3c shows the ratio of the magnitudes, *A*_2_/*A*_1_, where *A*_1_ and *A*_2_ are the magnitudes of Peaks 1 and 2, respectively. Clearly the ratio starts to increase around 50 °C, and increases exponentially up to 90 °C as shown by the inset of Figure 3c.

**Figure 4:**
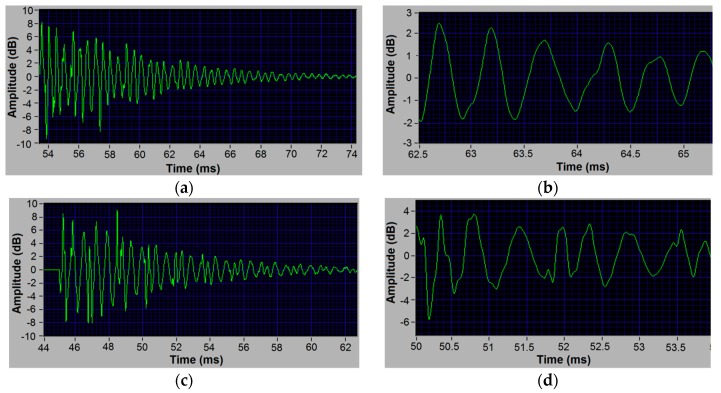

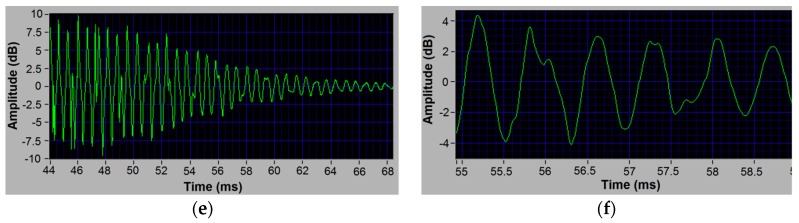
The original time-domain signal of specimen vibration. (**a**) The signal at 25 °C and (**b**) the corresponding enlarged image; (**c**) the signal at 75 °C and (**d**) the corresponding enlarged image; (**e**) the signal at 82 °C and (**f**) the corresponding enlarged image.

Considering that the frequency spectrums were obtained by the FFT of time-domain signals, we further analyzed the time-domain vibrational signals at 25, 75, and 82 °C. It can be seen that the vibrational amplitude at 25 °C (Figure 4a,b) decreases gradually due to internal frictions. The vibrational period (vibration duration in one cycle) is very stable, around 0.51 ms. Its reciprocal is the corresponding vibrational frequency 1960 Hz which matches Peak 1 (Figure 2a). At 75 °C, some peaks with short period appear in the vibrational signal (see Figure 4c and the enlarged image in Figure 4d), indicating that multiple vibration modes have been activated, interacting with each other. This complex signal leads to multiple large peaks in the frequency spectrum after FFT, as shown in Figure 2c. This interphase signal leads to two peaks in the frequency spectrum after FFT, as shown in Figure 2c. At 82 °C, the short-period signal segment disappears and the vibrational signal period is about 0.71 ms (see Figure 4e and the enlarged image in Figure 4f), corresponding to the peak around 1410 Hz in Figure 2e.

## 4. Discussion

When a simply supported beam is excited by an impact bar, the major response should be the bending vibration of the first-mode. The corresponding frequency is determined by the Young’s modulus, mass, and geometries of the specimen as shown in Equation (1). Therefore, there should only be one dominant peak in the frequency spectrum. With increasing the temperature, Young’s modulus in most materials would decrease due to thermal expansion [28,29] and thus the natural frequency should also decrease. Apparently, PMMA does not follow this trend. First, multiple peaks appeared in the frequency spectrum, which means that multiple vibrational modes were activated. Secondly, the relative amplitude of Peak 1 and Peak 2 changed with the temperature, indicating that different vibrational modes competed with each other and the dominant mode was changing with temperature. This raises two questions:

(1) What are these vibrational modes and why do they change with temperature?
(2) Can one still obtain the elastic properties of the material from the dominant peaks?

### 4.1. Possible Vibrational Mode of Peak 2

According to the beam vibration theory, the most possible vibration mode of Peak 2 is high-order bending modes, torsional modes, or both. The *n*th-order bending vibration frequency of a simply supported elastic beam can be expressed as

(2) $$f_{n}=\frac{k_{n}^{2}}{2\pi }\sqrt{\frac{EI}{\rho A}}\;$$

where *I* is the second moment of area of the cross section; *ρ* is density; *A* is the cross section area of the beam; *k_n_* is nth-order vibration wavenumber and *k*_2_ = 7.8532/L and *k*_3_ = 10.9956/L, in which *L* is the beam length. Using *E* = 6.43 GPa obtained above, one can find that the second-order and third-order frequencies of the PMMA specimen are 5416 and 10,613 Hz, respectively, which are much larger than the frequencies of the dominant peaks shown in Figure 2. Similarly, the torsional vibration frequency can be calculated by

(3) $$f_{n}^{T}=\frac{n}{2L}\sqrt{\frac{G\gamma }{\rho J_{P}}}\;$$

where *G* is shear modulus, γ is a torsional constant, and *J*_p_ is the polar moment of area of the cross section. The calculated first-order torsional frequency is 3505 Hz, which is inconsistent with the measured value in Figure 2. Moreover, these high-order bending modes and torsional mode would not change with temperature significantly, and cannot become the dominant vibrational mode at high temperature. Therefore, there should be some other mechanisms behind.

### 4.2. Structure Relaxation of PMMA

**Figure 5:**
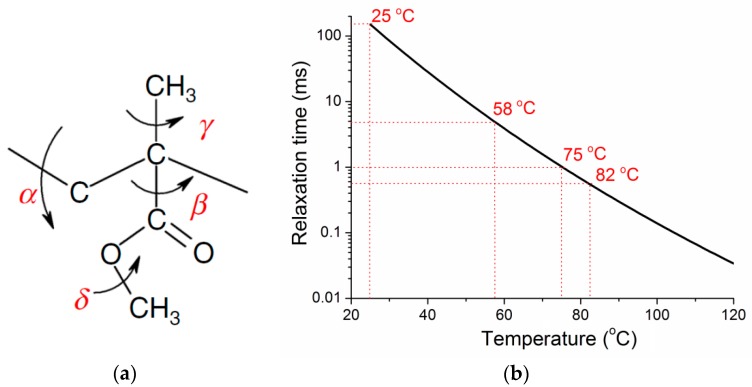
(**a**) The unit structure of PMMA; (**b**) The changes of β-relaxation time with temperature.

The property changes of a material are determined by its underlying microstructures. The basic structural unit of PMMA [30] includes two carbon atoms as the backbone, and two side groups (–CH_3_ and –COOCH_3_), as shown in Figure 5a. It was reported that the structural relaxation of PMMA can be divided into four major types relating to different molecular motions [30], i.e., α-relaxation (backbone rotation), β-relaxation (rotation of side group –COOCH_3_), γ-relaxation (rotation of side group –CH_3_), and δ-relaxation (rotation of –CH_3_ in the side group of –COOCH_3_). The α-relaxation normally happens during glass transition and should not affect the properties of PMMA below *T*_g_. γ- and δ-relaxations are very fast at room temperature compared to the vibration frequency and could not affect the properties of PMMA above 0 °C. Therefore, the only possible structural reason that causes the multiple vibrational frequencies and their changes with temperature should be the β-relaxation, which normally happens near room temperature.

The β-relaxation in PMMA is attributed to the hindered rotation of the –COOCH_3_ side group about the C–C bond that connects it to the main chain (backbone) [31]. The character time of β-relaxation of PMMA can be expressed as [32]:

(4) $$\tau_{\beta }=\tau_{0}\exp(E_{\beta }/RT)\;$$

where *τ*_0_ is the reference time, *E*_β_ is the active energy of relaxation, and *R* is the gas constant number (8.314 J·mol^−1^·K^−1^). The parameters determined by dielectric relaxation measurement in Reference [32] are *τ*_0_ = 10^−15.9^ s, *E*_β_ *=* 86 kJ·mol^−1^. According to this equation, one can get the relaxation time as a function of temperature as shown in Figure 5b. It is noted that the relaxation time at 25 °C is about 150 ms, which is much larger than the vibrational period. Therefore, at room temperature the effect of β-relaxation on the vibration frequency of the specimen is very small. At 58 °C, the relaxation time is about 4.7 ms, which is an order higher than the vibrational period. The effect of β-relaxation starts to appear and increases with temperature, as observed in Figure 2b. At 75 °C, the relaxation time is about 1 ms, which has the same order of the vibrational period and should have significant effect on the vibration. At 82 °C, the relaxation time is about 0.568 ms, which is smaller than the vibrational period (0.71 ms). In this case, the vibrational motions of the –COOCH_3_ side group could be relaxed in one vibrational period, and thus cannot vibrate together with the backbone chain.

Based on the above analysis, the formation mechanism of the multiple peaks in the vibrational frequency spectrum of PMMA can be summarized as follows. The vibration of PMMA near room temperature can be divided into two modes: overall microstructure vibration and partial microstructure vibration after β-relaxation. At a low temperature, the stiffness of sub-microstructures formed by entanglement of side groups is large enough so that they can vibrate together with the main chains. However, with increasing the temperature, the second bond starts to fail due to β-relaxation, making the entanglement effect weaker. The sub-microstructure cannot follow the vibration of the main chains, and thus the vibration mode of the backbone network with a lower frequency is enhanced gradually. Finally, the dominated vibration mode becomes the first-order bending vibration of PMMA after structure relaxation, evidenced by the dominated Peak 2 at high temperature (Figure 2e,f). The spectra in Figure 2 do have many other peaks. However, the amplitudes of them are very small compared to the first two and never become dominant during the whole heating/cooling process. Therefore, it is not necessary and also very difficult to find their corresponding vibrational modes.

### 4.3. The Modulus of PMMA Network

The above analysis indicates that the frequencies of Peaks 1 and 2 should correspond to the vibrations of the overall microstructure and the microstructure after relaxation, respectively. According to Equation (1), their corresponding moduli are 6.43 and 3.5 GPa, respectively. Many Young’s moduli of PMMA reported in the literature are about 3 GPa [30]. This is because most of these values were measured by quasi-static tensile/compressive tests, in which the character time of the β-relaxation is much smaller than the loading time.

Considering that the frequencies of both peaks increase with temperature as shown in Figure 3a, the corresponding network moduli should also increase with temperature. According to the first law of thermal dynamics, the change in internal energy of a polymer network, d*U*, is the summation of all the energy changes, i.e.,

(5) dU=TdS−pdV+FdL 

where *T*d*S* is the heat added to the system, −*p*d*V* is the work done to change the network volume, and *F*d*L* is the work done to change the shape of the network. According to the Maxwell relationship, ${\left(\partial S/\partial L\right)}_{T,V}=-{\left(\partial F/\partial T\right)}_{T,L}$, the force *F* applied on the network consists of two contributions [33]:

(6) $$F={\left(\partial U/\partial L\right)}_{T,V}+T{\left(\partial F/\partial T\right)}_{V,L}\;$$

In a polymer glass above *T*_g_, the deformation is mainly achieved by entropy change (entropy elasticity theory) and the overall modulus can be evaluated by ignoring the contribution of internal energy. In a polymer glass below *T*_g_, however, both the contributions of internal energy and entropy should be considered because of the secondary structure relaxation (𝛽-relaxation in PMMA). At a given deformation *L*, if temperature increases, stress should also increase due to the increase of entropy, leading to modulus increase as well [33].

It should be noted that the network moduli discussed here is different from the overall modulus of the material. At both low and high temperatures, the measured modulus from the dominant peak can be regarded as the overall modulus. In the transition region, however, the overall modulus should be a combination of the moduli derived from both peaks [34,35], which will be elaborated in Section 4.4.

### 4.4. Young’s Modulus of Bulk PMMA Material

The two moduli obtained above cannot simply represent the Young’s modulus of the bulk PMMA. Following the Ashby’s idea [30] that relaxation in polymers requires the breakage of the secondary bonds, Mahieux et al. [34] developed modulus model which relates the instantaneous modulus of a polymer to temperature. This model assumes that any transition in the polymer requires the breakage of the secondary bonds. Considering the different nature of bonds and the heterogeneity of molecular arrangement, the failure of these bonds follows the Weibull distribution. Richeton et al. [35] further expanded this model by considering the strain rate effect. The modulus change of a polymer with temperature and frequency *f* can be expressed as:

(7) $$E(f)=(E_{1}(f)-E_{2}(f))\exp(-{\left(\frac{T}{T_{\beta }(f)}\right)}^{m_{1}})+(E_{2}(f)-E_{3}(f))\exp(-{\left(\frac{T}{T_{g}(f)}\right)}^{m_{2}})+E_{3}(f)\exp(-{\left(\frac{T}{T_{f}(f)}\right)}^{m_{3}})\;$$

where *E*_1_, *E*_2_, and *E*_3_ are the frequency-dependent moduli before β-relaxation, before α-relaxation (glass transition), and before fluidic flow, respectively; *T*_β_(*f*), *T*_g_(*f*), and *T*_f_(*f*) are the corresponding β-relaxation temperature, glass transition temperature, and flow temperature at a particular frequency *f*, respectively; and *m*_1_, *m*_2_, and *m*_3_ are statistical parameters. The strain rate dependence of modulus is given by [35]:

(8) $$E_{i}=(E_{i}^{\mathrm{ref}})(1+s\log(\frac{f}{f^{\mathrm{ref}}}))\;$$

where *i* is 1, 2, or 3; $E_{i}^{\mathrm{ref}}$ represents the Young’ s modulus at reference frequency *f*^ref^; and s is a constant (s = 0.078 for PMMA) [35]. According to the physical meaning of the parameters, it is interesting to see that *E*_1_ and *E*_2_ in this model should correspond to the measured moduli of the overall microstructure and the microstructure after β-relaxation, respectively. Recent theoretical works on the dynamics of polymer network also revealed the relationship between the relaxation-induced microstructure and modulus changes [36,37]. Therefore, this study provides a convenient and physically sound method to determine the parameters.

Richeton et al. [35] found that, the model can predict the Young’s modulus changes of PMMA measured by DMA with *E*_1_ = 8.6 GPa and *E*_2_ = 3.6 GPa at 1 Hz and by compressive tests with *E*_1_ = 5.9 GPa and *E*_2_ = 2.7 GPa at 25 Hz. Based on Equation (8), one can calculate the corresponding values of *E*_1_ at 2000 Hz and *E*_2_ at 1500 Hz, and compare them with the values obtained in this study (see Table 1). For both *E*_1_ and *E*_2_, the value obtained by IET is smaller than that of DMA tension test and larger than that of the compressive test. It is noted that the tensile modulus found in typical polymer databases is usually larger than the compressive modulus [35]. Considering that the bending deformation involves both tensile and compressive deformations, it is reasonable to have a medium value.

## 5. Conclusions

This paper reported notable multi-peak vibrational frequency spectrums of poly(methyl methacrylate) (PMMA) specimen under impulse excitation. At room temperature, the frequency of the dominant peak is at 1950 Hz. With increasing the temperature, a peak with a smaller frequency increases significantly and becomes dominant at temperatures above 82 °C. This changeover is identified to be the effect of the β-relaxation, and the frequencies of the two peaks are recognized as the natural vibrational frequencies of the overall microstructure and the microstructure after *β*-relaxation, respectively. The corresponding moduli of polymer glass microstructures are therefore determined, enabling the prediction of the Young’s modulus of PMMA over a wide range of temperature and strain rate.

## Figures and Tables

**Table 1:** The values of *E*_1_ at 2000 Hz and *E*_2_ at 1500 Hz.

Modulus	DMA (Tensile)	Uniaxial Compressive	IET (Bending)
*E*_1_ at 2000 Hz	11.14 GPa	5.9 GPa	6.43 GPa
*E*_2_ at 1500 Hz	4.6 GPa	3.14 GPa	3.5 GPa

## References

[1] Richeton J., Ahzi S., Vecchio K.S., Jiang F.C., Adharapurapu R.R. (2006). Influence of temperature and strain rate on the mechanical behavior of three amorphous polymers: Characterization and modeling of the compressive yield stress. Int. J. Solids Struct..

[2] Smith K.E., Parks S.S., Hyjek M.A., Downey S.E., Gall K. (2009). The effect of the glass transition temperature on the toughness of photopolymerizable (meth)acrylate networks under physiological conditions. Polymer.

[3] Hoy R.S. (2011). Why is understanding glassy polymer mechanics so difficult?. J. Polym. Sci..

[4] Fleischhauer R., Dal H., Kaliske M., Schneider K. (2012). A constitutive model for finite deformation of amorphous polymers. Int. J. Mech. Sci..

[5] Berdichevsky V.L., Herman J.N. (2016). On rheology of cross-linked polymers: 1. Slippage of polymer chains and its macroscopic modeling. Int. J. Eng. Sci..

[6] Mahieux C.A., Reifsnider K.L. (2002). Property modeling across transition temperatures in polymers: Application to thermoplastic systems. J. Mater. Sci..

[7] Sujithra R., Srinivasan S.M., Arockiarajan A. (2014). Modeling memory effects in amorphous polymers. Int. J. Eng. Sci..

[8] Schmidt-Rohr K., Kulik A.S., Beckham H.W., Ohlemacher A., Pawelzik U., Boeffel C., Spiess H.W. (1994). Molecular nature of the beta-relaxation in poly(methyl methacrylate) investigated by multidimensional NMR. Macromolecules.

[9] Smith G.D., Bedrov D. (2007). Relationship between the alpha- and beta-relaxation processes in amorphous polymers: Insight from atomistic molecular dynamics simulations of 1,4-polybutadiene melts and blends. J. Polym. Sci..

[10] Fytas G., Wang C.H., Fischer E.W., Mehler K. (1986). Evidence of two relaxation processes in the photon-correlation spectra of poly(methyl methacrylate) above Tg. J. Polym. Sci..

[11] Garwe F., Schonhals A., Lockwenz H., Beiner M., Schroter K., Donth E. (1996). Influence of cooperative alpha dynamics on local beta relaxation during the development of the dynamic glass transition in poly(n-alkyl methacrylate)s. Macromolecules.

[12] Muzeau E., Perez J., Johari G.P. (1991). Mechanical spectrometry of the beta-relaxation in poly(methyl methacrylate). Macromolecules.

[13] Anand L., Ames N.M., Srivastava V., Chester S.A. (2009). A thermo-mechanically coupled theory for large deformations of amorphous polymers. Part I: Formulation. Int. J. Plast..

[14] Deng Y.J., Peng L.F., Lai X.M., Fu M.W., Lin Z.Q. (2017). Constitutive modeling of size effect on deformation behaviors of amorphous polymers in micro-scaled deformation. Int. J. Plast..

[15] Srivastava V., Chester S.A., Ames N.M., Anand L. (2010). A thermo-mechanically-coupled large-deformation theory for amorphous polymers in a temperature range which spans their glass transition. Int. J. Plast..

[16] Basterra-Beroiz B., Rommel R., Kayser F., Westermann S., Valentin J.L., Heinrich G. (2018). Swelling of polymer networks with topological constraints: Application of the Helmis-Heinrich-Straube model. Express Polym. Lett..

[17] Heidemann K.M., Sharma A., Rehfeldt F., Schmidt C.F., Wardetzky M. (2015). Elasticity of 3D networks with rigid filaments and compliant crosslinks. Soft Matter.

[18] Meng F.L., Terentjev E.M. (2017). Theory of semiflexible filaments and networks. Polymers.

[19] Chen W., Lu F., Cheng M. (2002). Tension and compression tests of two polymers under quasistatic and dynamic loading. Polym. Test..

[20] Narva K.K., Lassila L.V., Vallittu P.K. (2005). The static strength and modulus of fiber reinforced denture base polymer. Dent. Mater..

[21] Davis W.M., Macosko C.W. (1978). Non-linear dynamic mechanical moduli for polycarbonate and pmma. J. Rheol..

[22] Ornaghi H.L., Bolner A.S., Fiorio R., Zattera A.J., Amico S.C. (2010). Mechanical and dynamic mechanical analysis of hybrid composites molded by resin transfer molding. J. Appl. Polym. Sci..

[23] Montecinos S., Tognana S., Salgueiro W. (2016). Determination of the Young’s modulus in CuAlBe shape memory alloys with different microstructures by impulse excitation technique. Mater. Sci. Eng..

[24] Tognana S., Salgueiro W., Somoza A., Marzocca A. (2010). Measurement of the Young’s modulus in particulate epoxy composites using the impulse excitation technique. Mater. Sci. Eng..

[25] Liu W.D., Liu M., Zhang L.Z. (2016). Oxidation-induced mechanical deterioration and hierarchical cracks in glassy carbon. Carbon.

[26] Liu W.D., Ruan H.H., Zhang L.Z. (2014). Revealing structural relaxation of optical glass through the temperature dependence of Young’s Modulus. J. Am. Ceram. Soc..

[27] Lei Y., Adhikari S., Friswell M.I. (2013). Vibration of nonlocal Kelvin-Voigt viscoelastic damped Timoshenko beams. Int. J. Eng. Sci..

[28] Anderson O.L. (1966). Derivation of wachtmans equation for temperature dependence of elastic moduli of oxide compounds. Phys. Rev..

[29] Wachtman J.B., Tefft W.E., Lam D.G., Apstein C.S. (1961). Exponential temperature dependence of Young’s modulus for several oxides. Phys. Rev..

[30] Jones D.R.H., Ashby M.F. (2011). Engineering Materials 1—An Introduction to Properties, Applications and Design.

[31] Bergman R., Alvarez F., Alegria A., Colmenero J. (1998). The merging of the dielectric alpha- and beta-relaxations in poly(methyl methacrylate). J. Chem. Phys..

[32] Casalini R., Snow A.W., Roland C.M. (2013). Temperature dependence of the Johari-Goldstein relaxation in poly(methyl methacrylate) and poly(thiomethyl methacrylate). Macromolecules.

[33] Weiner J.H. (2002). Statistical Mechanics of Elasticity.

[34] Mahieux C.A., Reifsnider K.L. (2001). Property modeling across transition temperatures in polymers: A robust stiffness-temperature model. Polymer.

[35] Richeton J., Schlatter G., Vecchio K.S., Remond Y., Ahzi S. (2005). A unified model for stiffness modulus of amorphous polymers across transition temperatures and strain rates. Polymer.

[36] Jurjiu A., Biter T.L., Turcu F. (2017). Dynamics of a polymer network based on dual sierpinski gasket and dendrimer: A theoretical approach. Polymers.

[37] Jurjiu A., Galiceanu M. (2018). Dynamics of a polymer network modeled by a fractal cactus. Polymers.

